# Peroxisome Proliferator-Activated Receptor α in Lipoprotein Metabolism and Atherosclerotic Cardiovascular Disease

**DOI:** 10.3390/biomedicines11102696

**Published:** 2023-10-03

**Authors:** Elena Valeria Fuior, Evangelia Zvintzou, Theodosios Filippatos, Katerina Giannatou, Victoria Mparnia, Maya Simionescu, Anca Violeta Gafencu, Kyriakos E. Kypreos

**Affiliations:** 1 Institute of Cellular Biology and Pathology, “Nicolae Simionescu” of the Romanian Academy, 050568 Bucharest, Romania; elena.fuior@icbp.ro (E.V.F.); liliazv@upatras.gr (E.Z.); maya.simionescu@icbp.ro (M.S.); 2 Pharmacology Laboratory, Department of Medicine, University of Patras, 26500 Rio Achaias, Greece; katerinagiannatou001@gmail.com (K.G.); victoriamparnia2@gmail.com (V.M.); 3 Internal Medicine Clinic, Department of Medicine, University of Crete, 71500 Heraklion, Greece; filtheo@gmail.com; 4 Department of Life Sciences, School of Sciences, European University Cyprus, 2404 Nicosia, Cyprus

**Keywords:** peroxisome proliferator-activator receptor, lipoproteins, atherosclerosis, coronary heart disease, pharmacology

## Abstract

Peroxisome proliferator-activated receptors (PPARs) are a group of ligand-binding transcription factors with pivotal action in regulating pleiotropic signaling pathways of energetic metabolism, immune responses and cell proliferation and differentiation. A significant body of evidence indicates that the PPARα receptor is an important modulator of plasma lipid and lipoprotein metabolism, with pluripotent effects influencing the lipid and apolipoprotein cargo of both atherogenic and antiatherogenic lipoproteins and their functionality. Clinical evidence supports an important role of PPARα agonists (fibric acid derivatives) in the treatment of hypertriglyceridemia and/or low high-density lipoprotein (HDL) cholesterol levels, although the effects of clinical trials are contradictory and point to a reduction in the risk of nonfatal and fatal myocardial infarction events. In this manuscript, we provide an up-to-date critical review of the existing relevant literature.

## 1. Introduction

Peroxisome proliferator-activated receptors (PPARs) are a group of ligand-binding transcription factors with pivotal action in regulating pleiotropic signaling pathways of energetic metabolism, immune responses and cell proliferation and differentiation [1,2,3,4,5]. The three members—PPARα, PPARβ/δ and PPARγ—structurally belong to the subfamily 1 group C of the nuclear receptor (NR) superfamily, and hence they are alternatively named NR1C1, NR1C2 and NR1C3, respectively. Initially identified as xenobiotic-induced molecules with the consequent expansion of the peroxisomes [6], they became intensively studied from a therapeutic perspective as fibrates lowered plasma lipid levels via PPARα, while thiazolidinediones (glitazones) promoted insulin sensitization via PPARγ [7]. Unfortunately, besides the metabolic improvements, these ligands also provoke unwanted side effects. Thus, the ultimate goal of pharmacological studies would be the development of selective PPAR modulators (SPPARMs) with a suitable activation profile for the treatment of dyslipidemia and type 2 diabetes. To this aim, extensive research is ongoing for thorough understanding of the molecular mechanisms involved in PPAR-mediated transcriptional regulation [8].

**Figure 1:**
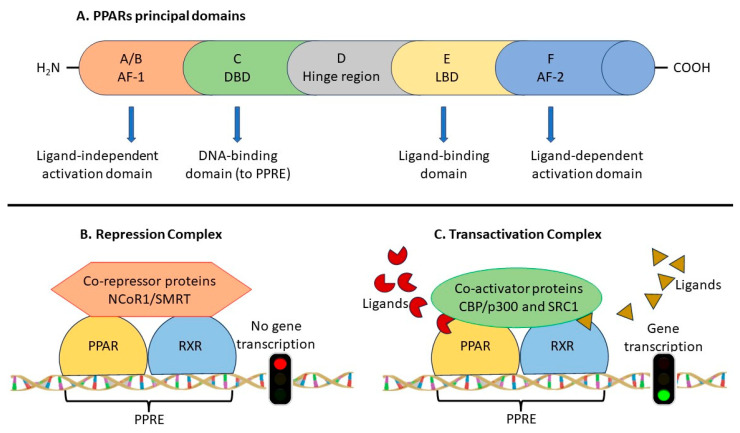
Schematic representation of the principal domains of PPARs (**A**) and the repression (**B**) and transactivation (**C**) complexes. PPAR: peroxisome proliferator-activated receptor; PPRE: PPAR response element; AF: activation factor; DBD: DNA-binding domain; LBD: ligand-binding domain; NCoR1: nuclear receptor corepressor 1; SMRT: silencing mediator of retinoic acid and thyroid hormone; CBP/p300: CREB-binding protein; SRC1: steroid receptor coactivator 1; RXR: retinoid X receptor.

PPARs have similar amino acid sequences and, as all NR, possess a modular architecture, based on functional domains [9,10]. Thus, they comprise five parts: (A/B) the N-terminal region containing a ligand-independent transactivation function (AF-1), (C) the DNA-binding domain (DBD), (D) a flexible hinge, (E) the ligand binding domain (LBD) and (F) the C-terminal region with the ligand-dependent activation domain AF2 (Figure 1A). Of these, the DBD is the most conserved, followed by the LBD. Heterodimerization with the retinoid X receptor alpha (RXRα) is mandatory for DNA binding to occur [11]. The DBD contains two zinc finger motifs at its edges, which allow binding to the target genes at the peroxisome proliferator response elements (PPRE), a tandem repeat of the AGG(A/T)CA sequence, separated by one (direct repeat 1) or two nucleotides (direct repeat 2). The hinge docks co-regulatory molecules (activators or repressors). The LBD is composed of thirteen alpha helices (H1-H12 and H2′) and a four-stranded beta-sheet. It contains a large Y-shaped lipid-binding pocket of 1200–1400 Å [3], which can accommodate various molecules, either endogenous or exogenous, natural or synthetic, through an induced fit mechanism [12,13]. The amino acid sequence of the pocket is less conserved and explains the selective action of ligands and the nonoverlapping effects of PPARs in intracellular signaling [14]. Of note, the affinity constants are in the nanomolar range for the exogenous ligands, but in the micromolar range for the endogenous molecules (reviewed by Lamas Bervejillo and Ferreira 2019 [15]), thus raising the possibility that PPARs could still be orphan receptors, whose cognate endogenous ligands remain to be found.

The modulatory effects of PPARs on various signaling cascades stem from three main mechanisms: (i) the ligand-independent repression, (ii) the ligand-dependent transactivation and (iii) the nontranscriptional ligand-dependent transrepression (reviewed by Daynes and Jones 2002 [3] and Lamas Bervejillo and Ferreira 2019 [15]).

The ligand-independent repression corresponds to the unliganded state of the PPAR–RXR heterodimer, which makes complexes with corepressor proteins such as the nuclear receptor corepressor 1 (NCOR1) and the silencing mediator of retinoic acid and thyroid hormone (SMRT) (Figure 1B). Worth mentioning, in the case of PPAR β/δ, the repressing assembly is able to bind DNA [16], and thus it may function to inhibit certain signaling effects of the other isotypes.

Transactivation occurs upon ligand binding, and, as a conformational change occurs, corepressors are replaced with coactivators such as the histone acetylases CREB-binding protein (CBP/p300) and steroid receptor coactivator 1 (SRC1), the complexed PPARs bind PPRE in the promoters of the target genes and RNA polymerase II initiates transcription (Figure 1C). The liganded state induced by various molecules promotes the recruitment of different coactivators and thus leads to tissue- and gene-selective effects. Moreover, the activation of certain genes is species-specific. This could be due to the lack of the corresponding cis-acting elements due to the presence of polymorphisms, as is the case for PPRE elements in the promoter of the *APOA1* gene in rats as compared to humans [17]. A remarkable difference is that PPARα activation induces hepatotoxicity in rodents, but not in humans, an effect that can be at least partially explained by the lower levels of PPARα in humans and may also raise attention to extrapolation of animal studies to humans [18]. Another explanation was that mice downregulate the tumor suppressor miRNA let-7C, while humans do not [19].

Transrepression is responsible for the anti-inflammatory effects of PPARs, as they interact with p65 and c-jun, sequestering the transcription factors NF-κB (nuclear factor-kB) and AP-1 (activator protein 1), respectively, and preventing downstream signaling in a DNA-independent manner [20,21].

An extensive analysis of tissue expression was performed in order to delineate the specific functions of each isoform. Thus, PPARα is correlated with fatty acid catabolism in tissues with high metabolic activity such as the liver, heart and muscle [14]. Nutrient availability controls the PPARα activity and fasting activates PPARα to help to maintain energetic homeostasis [22,23]. Moreover, PPARα mediates the diurnal responsiveness of cardiac and skeletal muscle to fatty acids [24].

While PPARα is involved in energy expenditure, PPARγ participates in energy storage. PPARγ exhibits two splice variants, the longer γ2 being expressed in adipose tissue and γ1 being found in immune cells. PPARγ2 is a master regulator of adipocyte differentiation and an insulin sensitizer upon glitazone binding [25]. PPARγ is also expressed in the urinary bladder and colon, and its dysfunction is associated with tumor development in these organs.

The function of PPARβ/δ is not so well defined, as it has low tissue specificity, being virtually ubiquitously transcribed. PPARβ/δ is involved in both lipid metabolism and insulin secretion [26]. As aforementioned, it can bind DNA while associated with corepressors, and thus it may dampen the effects of both PPARα and PPARγ. Nonetheless, similar to PPARα, PPARβ/δ enhances energy dissipation through enhanced fatty acid oxidation, whereas, resembling the effects of PPARγ, PPARβ/δ is an insulin sensitizer.

Post-translational modifications such as phosphorylation, SUMOylation, ubiquitination, acetylation and O-glycosylation play an important role in regulating PPARs activities [27], with effects on ligand binding, DNA binding, recruitment of coactivators and protein stability and degradation.

Protein kinases A (PKA) and C (PKC), mitogen-activated protein kinases (ERK- and p38-MAPK), AMP kinase (AMPK) and glycogen synthase kinase β (GSKβ) act on PPARs at various sites [28,29]. MAPK and CDK7 (cyclin-dependent kinase 7) increase the PPARα activity by phosphorylation of Ser12/Ser21. By contrast, phosphorylation of Ser73 by GSKβ leads to protein degradation. In PPARγ, confirmed phosphorylation sites are Ser112, Ser273 and Y78. Phosphorylation of Ser273 in PPARγ by CDK5 (cyclin-dependent kinase 5) is associated with obesity and insulin resistance and this finding spurred research toward developing small molecules to inhibit this modification.

Thus, it appears that the activities regulated by PPARs are controlled in a very complex network of interactions, involving not only ligands and PPRE sites but also other signaling cascades.

## 2. The Main Genes Involved in Lipid Metabolism Are Directly Regulated by PPARs

**Apolipoprotein A1** (APOA1) is the most abundant apolipoprotein present in high-density lipoprotein (HDL), playing an important role in various functions of HDL including reverse cholesterol transport, where free cholesterol is removed from peripheral tissues and shuttled to the liver for catabolism. Moreover, it is a cofactor for lecithin cholesterol acyltransferase which converts the free cholesterol to cholesteryl esters. The major source of APOA1 is the liver and the intestine, but it is also synthesized in low amounts by other tissues [30]. The *APOA1* gene is present in the *APOA1/C3/A4/A5* gene cluster located on chromosome 11 in humans and on chromosome 9 in mice. APOA1 has a well-established antiatherogenic role supported by many preclinical studies. For example, the *APOA1* transgenic mice on *LDLR* (low-density lipoprotein receptor) deficient background present resistance to diet-induced atherosclerosis [31].

The mechanism of the *APOA1* gene regulation is complex since its proximal promoter contains many nuclear factor binding sites [32,33]. Hormones, such as cortisol and retinoids increase the *APOA1* promoter activity, while interleukin-1β (IL-1β), tumor necrosis factor α (TNFα) or bisphenol A downregulate the *APOA1* gene expression [34,35]. PPARs also regulate the *APOA1* expression. An in silico analysis (https://molotool.autosome.org/ accessed on 13 February 2023) determined that the *APOA1* promoter presents two binding sites for PPARα/PPARγ and one for PPARγ (Table 1). The presence of the PPRE in the *APOA1* promoter was experimentally confirmed, at least in part. Using human hepatoma cells (HepG2) the PPARα-response element required for pioglitazone-induced modulation was determined to be located at −214/−192 bp, upstream of the transcription start site [36], overlapped with the binding site −186/−202 (Table 1) determined in silico. Fibrates and other PPARα ligands induce the upregulation of the human *APOA1* promoter [17]. The murine *APOA1* gene presents a 3-base variation in the PPARα binding site of the promoter inactivating this responsive element, and thus the murine gene cannot be upregulated by fibrates [37]. In the *APOA1* transgenic mice containing 5,5 kb *APOA1* promoter, the upregulation of the human *APOA1* gene by the fenofibrate induced a decrease in triglycerides level and an increase in HDL cholesterol (HDL-C); however, in the same model, gemfibrozil (another PPARα ligand) did not affect the transcriptional level of *APOA1*, probably because of the recruitment of different coactivators on the promoter [38]. The human *APOA1* promoter activity is downregulated by NF-κB [35,39]. Interestingly, NF-κB negative modulation on the *APOA1* expression was blocked by the overexpression of (NF-kB inhibitor alpha) IκBα but also by the selective inhibitor of PPARα, MK886, or by mutations of the PPRE of the *APOA1* promoter [39]. These data suggested that NF-κB can affect the *APOA1* gene expression by a direct mechanism but also through an indirect pathway that involves PPARα. 

**Apolipoprotein A2** (APOA2), the second most abundant apolipoprotein in HDL plays an important role in lipid metabolism but is involved in a variety of other processes such as plasma glucose homeostasis, amyloidosis and cancer [40,41]. Human APOA2 forms homodimers, due to the presence of a cysteine amino acid in the N-terminal end of the peptide, while murine APOA2 lacks this residue and is found in plasma as a monomer. Thus, the functions of APOA2 may differ significantly in humans compared to mice.

The human *APOA2* gene is located on chromosome 1, in the region 1q21-q23 [42]. The regulation of the human *APOA2* gene expression implies 14 regulatory elements located in the proximal promoter but also the transcription factor binding sites located in the distal region [43]. A series of nuclear receptors regulate the human *APOA2* expression: PPARα, PPARγ, RXR, RORα, T3Rβ, HNF4-α and SREBP-2, as recently reviewed [41]. Molotool in silico analysis identified PPARα binding sites in the human *APOA2* promoter (Table 1). The regulatory potential of the binding site located at −720/−736 was experimentally confirmed since Vu-Dac et al. found a PPARα–RXR binding site in the region −740/ −714 of the *APOA2* promoter [44]. Fibrates upregulate the human *APOA2* gene via RXR-PPARα; however, fenofibric acid, but not the other fibrates, decreased the rat *APOA2* expression [45]. 

**Apolipoprotein A5** (APOA5) is a minor component of HDL, but it is also found in very low-density lipoproteins (VLDL) and. It plays an important role in the modulation of plasma triglyceride levels, stimulating APOC2, lipoprotein lipase and triglyceride hydrolysis and inhibiting the hepatic VLDL-triglyceride production [46,47].

The *APOA5* gene is located in the *APOA1/C3/A4/A5* gene cluster on chromosome 11q23. The group led by Fruchart found that in human primary hepatocytes, the treatment with PPARα ligands (Wy 14,643 or fenofibrate) induces a strong upregulation of the *APOA5* gene. In addition, the group led by Rodriguez determined that in human HepG2 cells and primary hepatocytes isolated from cynomolgus liver GW9003 PPARα agonists increase the *APOA5* gene expression [48]. Both groups demonstrated by the experiments of deletion, gel shift analysis and mutagenesis that the PPRE located at the position −272/−260 is functional and responsive to the PPARα. This binding site is the same as that we determined in silico using Molotool analysis in the region −771/−787 relative to the transcription start of the variant 3 transcripts of the *APOA5* gene (Table 1). 

**Apolipoprotein C3** (APOC3) is a component of the chylomicrons, VLDL and HDL [49]. APOC3 is a small peptide (79 amino acids), synthesized and secreted by the liver and small intestine [50]. Because APOC3 is a strong inhibitor of triglycerides hydrolysis it became a target in various therapeutic approaches for hyperlipemia [51,52]. 

Several groups demonstrated that fibrates downregulate the *APOC3* expression. Staels and co-authors showed that fibrates inhibit the activity of −1415/+24 *APOC3* promoter in HepG2 cells [53], in concordance with the sites that we found on the *APOC3* promoter by the in silico analysis (Table 1). Moreover, Hertz and collaborators demonstrated that the PPAR binding site located −87/−66 in the human *APOC3* promoter is active; moreover, the binding of PPAR to this specific binding site, displaces HNF4 from the *APOA3* promoter and thus suppresses the *APOC3* transcription. 

A study on a small number of patients showed that a fat-restricted diet and treatment with 300 mg of fenofibrate per day for one month reduced the levels of total cholesterol, triglycerides, APOC2 and APOC3 [54]. Fenofibrate at 200 mg/d also reduced the APOC2 and APOC3 levels in subjects with the metabolic syndrome [55]. A recent multinational, randomized, double-blind, controlled clinical trial on a cohort of patients with type 2 diabetes who received 0.2 mg pemafibrate twice daily, showed that pemafibrate reduced the levels of APOC3 by 27.6% [56].

**Apolipoprotein E** (APOE) is involved in a series of processes, among which lipid metabolism is an important one since it is a component of almost all lipoprotein classes. APOE, a 35 kDa glycoprotein, is expressed mainly in the liver, but there are also other minor sources such as macrophages, adipocytes and astrocytes. Due to its multiple interactions with lipids and various proteins (i.e., LDL receptor, β-amyloid), APOE is involved in various pathophysiological processes as we recently reviewed [57]. The human *APOE* gene is located on chromosome 19 at the 5′end of a cluster containing also *APOC1*, *APOC2* and *APOC4* genes. The *APOE* gene regulation is complex and involves not only the proximal promoter but also tissue-specific distal regulatory elements [58]. In macrophages, the *APOE* gene is downregulated by NF-κB [59] and is upregulated by the glucocorticoid receptors [60] acting on the promoter, while the signal transducer and activator of transcription 1 (STAT1) acts on the distal regulatory element, the multienhancer 2 (ME2) [61]. 

The in silico analysis revealed a PPRE in the *APOE* promoter (Table 1), but we could not confirm its functionality, using RAW264.7 macrophages. However, the group led by Mazzone demonstrated that, in adipocytes, the *APOE* expression is upregulated by pioglitazone, a PPARγ agonist. Moreover, they showed that ciglitazone, another PPARγ agonist did not regulate the expression of *APOE* in macrophages but modulated it in 3T3-L1 adipocytes. Mazzone’s group demonstrated that the PPRE is located in the multienhancer [62]. The PPARγ site found in silico in ME2 has very low affinity. 

**Apolipoprotein M** (APOM) plays an important role in HDL metabolism, being mainly expressed in liver and kidney [63]. APOM is the chaperon for sphingosine-1-phosphate, a bioactive sphingolipid [64]. The *APOM* gene is modulated by various transcription factors. Liver receptor homolog-1 (LRH-1) [65], forkhead box A2 (Foxa2) [66], hepatocyte nuclear factor 4 (HNF-4) [67], hepatocyte nuclear factor 1 (HNF-1), JunB and c-Jun [68] are some of the transcription factors that regulate the *APOM* promoter.

The group led by Kardassis uncovered the hormone–response element (HRE) in the *APOM* promoter (−33/−21), corresponding to the situs found by in the silico analysis (Table 1). This situs is functional and can bind retinoid X receptor (RXR) homodimer or heterodimers with thyroid hormone receptor, liver X receptor (LXR) and PPARα [67]. Kurano et al. demonstrated that PPARγ ligands increased the *APOM* expression and S1P in HepG2 cells; moreover, the treatment with pioglitazone which suppressed PPARγ decreased the *APOM* and S1P levels in diet-induced obese mice [69].

**Lipoprotein lipase** (LpL) hydrolyzes triglycerides to glycerol and free fatty acids which are provided to the peripheral tissues and used as an energy source or stored. LpL is synthesized in the heart, adipose tissue, muscle, macrophage and other tissues [70]. After secretion, LpL is attached to the cell surface using a heparan sulfate proteoglycans anchor. Glycosylphosphatidylinositol-anchored high-density lipoprotein binding protein 1 (GPIHBP1) transports LpL from the cell surface to the capillary endothelium where it hydrolyzes the triglyceride moieties of chylomicrons and VLDL [71,72]. The LpL activity is positively modulated by APOC2 and APOA5; by contrast, APOC3 and angiopoietin-like proteins 3, 4 and 8 inhibit LpL activity, and thus therapeutic targeting of LpL includes the *LpL* gene regulation as well as modulation of the proteins that modulate the LpL activity [73]. 

In humans, the *LpL* gene is located on chromosome 8. The *LpL* gene contains numerous regulatory elements such as PPRE, sterol regulatory element 2, oxysterol liver X receptor responsive element, interferon-γ responsive element, nuclear factor-1-like motif and AP-1 binding site. 

It was shown that fibrates stimulate the *LpL* gene expression thus increasing lipolysis and affecting triglyceride (TG) metabolism [50,74]. Experiments performed in vitro or in vivo (in rats) revealed that PPARα ligands induced the *LpL* expression in hepatocytes, while PPARγ ligands did not modulate the hepatic *LpL* expression but induced the expression of *LpL* in adipose tissue [75]. The human *LpL* promoter contains PPRE, as determined by the in silico analysis (Table 1). It was experimentally demonstrated (by methylation interference and gel retardation assay) that this regulatory element is functional, binds PPARα/RXR or PPARγ/RXR heterodimers and thus is responsive to fibrates and thiazolidinediones [76]. 

**Cholesteryl ester transfer protein** (CETP) plays an important role in cholesterol homeostasis, being involved in the transfer of the cholesteryl esters from HDL to apolipoprotein B-containing lipoproteins [77]. Mice lack CETP and thus are protected from atherosclerosis, while in transgenic mice expressing increasing levels of CETP, lower levels of HDL and higher levels of chylomicrons, VLDL and LDL were reported [78]. On the other hand, studies revealed that in the absence of CETP, reverse cholesterol transfer is attenuated [79]. 

The human *CETP* gene is located in chromosome 16, Cytogenetic band 16q13. The *CETP* gene expression is modulated by inflammatory stress factors, hormones and diet. It was also shown that LXR and FXR [80] upregulate the *CETP* expression, while glucocorticoids downregulate the *CETP* expression [81].

Our in silico analysis revealed a PPRE in the region −675/−691 (Table 1). Cheema et al. revealed a functional PPRE in the *CETP* promoter −413/−401, which led to increased *CETP* promoter activity [82] under 25-OH cholesterol treatment [83]. There are also other data showing that fibrates increase the CETP activity when others reported downregulation of *CETP* by PPARα ligand [83,84].

Thus, the current data are not conclusive regarding the role of PPAR in the CETP regulation. The differences are emerging because various PPAR ligands and different models were used.

**Scavenger receptor class B type 1** (SRB1) transporter mediates the cholesterol transport (export and uptake). It was demonstrated that SRB1 facilitates cholesterol efflux through interactions with the lipids bound to APOE [85].

The human and rat *SRB1* promoter contains PPREs, and thus oxysterols and fibrates modulate its transcription [86,87,88]. The fibrate-treated mice had larger HDL particles possibly due to the upregulation of phospholipid transfer protein and downregulation of *SRB1* [38]. Interestingly, fenofibrate enhances SRB1 degradation in a post-endoplasmic reticulum compartment, without the involvement of the proteasome, calpain protease or the lysosome.

On the other hand, the PPAR modulation pathway is affected by various other transcription factors and signaling molecules. It was demonstrated that HNF-4 enhances the PPARγ-mediated *SRB1* gene transcription [86,89], while the modulation induced by Ras/MEK/ERK signaling is intermediated by PPARα-inducible degradation pathways [90]. The interaction of ERK signaling with PPAR was also demonstrated using a recombinant antibody that selectively activates ERK1/2, which upregulated the *SRB1*, *APOA1* and *APOA2* gene expression [91]. Mutation performed in PPARα/RXR binding site found on the *SRB1* promoter abolished the *SRB1* promoter activity modulation induced by the ERK activation.

## 3. Genes Involved in Lipid Metabolism Are Indirectly Regulated by PPARs

**ATP Binding Cassette Transporter A1** (ABCA1) is a key protein in the de novo biogenesis of HDL, but it also facilitates the efflux of cholesterol from macrophages [92,93,94,95]. The human *ABCA1* gene is located on chromosome 9 region q31.1. PPAR agonists (13-hydroxy linoleic acid and pioglitazone) induced the *ABCA1* gene expression in macrophages [96,97]. However, data suggest that these effects are indirect and most probably mediated by LXRα which induces the *ABCA1* promoter transcription [98]. PPARδ activators appeared to induce the *ABCA1* expression and cholesterol efflux moderately and to increase the HDL levels in an obese monkey model [99].

**Proprotein convertase subtilisin/kexin type 9** (PCSK9) plays an important role in lipid metabolism, promoting LDLR degradation in hepatocytes. In the proximal promoter, no PPREs were identified by the in silico analysis. However, a clinical study showed that plasma PCSK9 concentrations were correlated with LDL cholesterol (LDL-C) and total cholesterol in patients with diabetes. Six-week treatment with fenofibrate decreased plasma PCSK9 concentrations [100].

## 4. PPARα in Atherosclerosis and Related Disorders

Atherosclerosis is a condition that is caused by the accumulation of plaque in the arterial wall, composed of lipids, inflammatory cells, smooth muscle cells and connective tissue. The development of atherosclerosis is associated with several other disorders of the metabolic syndrome such as dyslipidemia, insulin resistance, type 2 diabetes and obesity. Dyslipidemia is a condition characterized by elevated levels of triglyceride-rich lipoprotein particles, low levels of HDL-C and elevated levels of LDL-C [101].

Numerous studies have shown the beneficial effect of PPARα agonists on lipoprotein metabolism, inflammation and insulin resistance. It has been demonstrated that PPARα agonists cause browning of the white adipose tissue (WAT) [102], which reduces cytokine production and increases adiponectin synthesis [103], thus improving systemic insulin resistance and inflammation [104]. Additionally, PPARα agonist therapy increased the expression of fatty acid oxidation enzymes, which consequently decreased hepatic TG levels [105]. PPARα agonists also promoted LPL activity, further reducing VLDL-TG levels [106]. In patients with concomitant hyperlipidemia, the well-known PPARα agonist fenofibrate reduced the postprandial increase in chylomicron remnants [107]. Additionally, by lowering intestinal chylomicron synthesis, fenofibrate significantly inhibited the postprandial increase in TG and APOB48 [108,109]. Fibrates have, also, been shown to elevate HDL-C levels by inducing the transcription of *APOA1* and *APOA2* [106]. Treatment with fibrates such gemfibrozil and fenofibrate of 8500 individuals with established coronary artery disease led to significant TG reduction and modest HDL raising effectiveness [110].

Though it is customary to believe that PPARα is the sole member of the PPAR family with antiatherosclerotic properties, it is worth mentioning that PPARγ agonists have been demonstrated to successfully reduce excessive plasma free fatty acid levels and improve excessive lipid accumulation in peripheral tissues such as the liver, the heart and the skeletal muscle. Furthermore, PPARγ agonists reduce hyperinsulinemia/insulin resistance, by modulating the expression of inflammatory cytokines and adipokines that affect muscle and hepatic metabolism and overall insulin sensitivity [111]. On top of reducing hyperglycemia and improving insulin action, pioglitazone or rosiglitazone treatment of individuals with type 2 diabetes (T2DM) was associated with remarkable improvements in plasma triglyceride and HDL-C levels and in LDL particle concentration and size [112,113,114,115].

Previous studies have suggested that either PPARα [98] or PPARγ [98,116] activation promotes HDL-mediated cholesterol efflux from macrophages by enhancing the expression of ABCA1. Furthermore, the expression of PPARα and/or PPARγ in several types of vascular cells, such as macrophages, endothelial cells and vascular smooth muscle cells, implies that direct vascular effects may be a factor in the effectiveness of possible antiatherosclerosis treatments [101]. Both PPARγ and PPARα agonists have been reported to produce a range of anti-inflammatory effects in vascular cells. In particular, either PPARα [117] or PPARγ [118,119,120] activation inhibits cytokine-induced vascular cell adhesion and suppresses monocyte–macrophage migration. The PPARα and PPARγ agonists, fibrates and thiazolidinediones (TZDs), respectively, are in clinical use for several decades as medications to treat dyslipidemia and hyperglycemia in patients with T2DM. Although fibrates and TZDs are particularly efficient in improving either dyslipidemia or insulin resistance, many of these agonists also show additional biological reactions and adverse effects. This has been attributed to target complexes that comprise a large number of coactivator and corepressor proteins related to target gene promoters [121]. 

These data suggest that the dual PPARα/γ agonism may simultaneously reduce atherogenic triglycerides, raise cardio-protective HDL levels and improve insulin resistance while overcoming off-target side effects. During the past decade, considerable efforts have been made to develop new highly PPAR-specific drugs [122]. Currently, two PPARα/γ agonists, saroglitazar and lobeglitazon, have been marketed and are now in clinical use in India and Korea, respectively [122]. Future research should focus on finding PPARα single, double or pan agonists with high selectivity and sensitivity while also limiting off-target activity, since PPARα is a master regulator of genes implicated in lipoprotein metabolism.

## 5. PPARα Pharmacology

Fibrates (ATC: C10AB) are fibric acid derivatives that serve as agonists (activators) of the nuclear receptor PPARα in hepatocytes. Given the complex role of fibrates in gene regulation and in particular in the regulation of genes associated with lipid and lipoprotein metabolism, the precise mechanism of their action is not fully elucidated. Their biochemical effects include an effective reduction in hepatic VLDL-triglyceride secretion and a substantial increase in the LpL activity. In addition, fibrates stimulate fatty acid oxidation in the liver and skeletal muscle. Moreover, fibrates exert direct anti-inflammatory effects on visceral fat and in the arterial wall [123]. As a result, their main pharmacological benefit is the effective reduction in plasma triglyceride levels along with an increase in plasma HDL-C levels. 

In terms of their pharmacokinetics, fibrates are in general well absorbed by the gastrointestinal tract, they associate with plasma proteins and are mainly excreted through the urine, either unchanged or in the form of glucuronide metabolites [124]. Fibrates are highly protein-bound drugs in vivo; thus, they have the potential to displace warfarin from its binding proteins and trigger an enhanced hypoprothrombinemic effect associated with prolonged bleeding. In addition, fibrates are mild to moderate inhibitors of CYP2C9 (Cytochrome P450 family 2 subfamily C member 9), which is the major enzymatic system responsible for warfarin metabolism, thus increasing effective warfarin levels and prolonging prothrombin time [125]. In patients with severely impaired hepatic and renal function, the use of fibrates is contraindicated. Rarely, side effects of fibrates may include myositis and rhabdomyolysis, mainly when administered in combination with a statin in elderly patients with many comorbidities or patients with impaired renal function [126,127]. Gemfibrozil is contraindicated to be combined with statins.

In everyday clinical practice (Table 2), fibrates are expected to result in a reduction in triglyceride levels up to 50% and an increase in HDL-C levels up to 20%; these effects are highly dependent on the corresponding baseline levels. Regarding LDL-C, a reduction of up to 20% may be observed, but in patients with high triglyceride levels, a paradoxically small LDL-C increase may be observed [128,129]. Fibrates may minimally affect LDL-C concentration, but they induce a shift from small dense LDL particles to larger buoyant LDL particles [130,131,132]. Small dense LDL particles predominate in patients with hypertriglyceridemia and insulin resistance, such as patients with diabetes, and increase the risk of atherosclerosis by regulating the activity of gene networks, impairing endothelial function and inducing inflammation [133]. Additionally, fibrates alter HDL particle distribution leading to larger HDL particles and improve inflammation-related parameters as well as many other atherosclerosis-related variables [134,135,136]. Fenofibrate also reduces uric acid concentration, which is considered a marker of atherosclerotic risk [137,138].

Numerous clinical trials have documented the role of fibrates currently used in clinical practice (bezafibrate, ciprofibrate, fenofibrate and gemfibrozil) in cardiovascular disease [123]. In the double-blind placebo-controlled Helsinki Heart Study, the administration of gemfibrozil for 5 years in 4081 asymptomatic middle-aged men (40–55 years) with primary dyslipidemia (non-HDL-C ≥ 200 mg/dL) reduced fatal and nonfatal myocardial infarction events by 34% and total cardiovascular events by 56%, with a more profound benefit for people with a body mass index (BMI) > 26 kg/m^2^ [139]. Notably, gemfibrozil reduced the incidence of coronary heart disease events by 71% in the high-risk subgroup with LDL-C/HDL-C ratio >5 and triglycerides concentration >203 mg/dL. In the double-blind placebo-controlled secondary prevention VA-HIT trial, which included 2531 men with coronary heart disease, HDL-C ≤ 40 mg/dL and LDL-C ≤ 140 mg/dL, gemfibrozil administration for a median follow-up of 5.1 years significantly reduced the composite endpoint of coronary heart disease death, nonfatal myocardial infarction or stroke by 24% and significantly reduced coronary heart disease events, stroke and coronary heart disease-related death by 22%, 25% and 22%, respectively [140]. In the double-blind placebo-controlled secondary prevention BIP trial, which included 3090 patients with a previous myocardial infarction or stable angina, total cholesterol (TC) of 180–250 mg/dL, HDL-C ≤ 45 mg/dL, triglycerides ≤ 300 mg/dL and LDL-C ≤ 180 mg/dL, bezafibrate administration for a mean of 6.2 years non-significantly reduced (−9%) the primary end point (fatal myocardial infarction, nonfatal myocardial infarction, sudden death). Notably, bezafibrate significantly reduced myocardial infarction by 39% and nonfatal myocardial infarction by 33% in patients with the metabolic syndrome, whereas it significantly reduced cardiac mortality by 66% in the subgroup of patients with four or five features of the metabolic syndrome [141]. It should be mentioned that these trials were conducted in the pre-statin era.

During the statin and low-LDL-C target era three trials have been conducted. The double-blind placebo-controlled FIELD trial included 9795 statin-naïve participants aged 50–75 years, with type 2 diabetes mellitus, a TC concentration of 116–251 mg/dL and a TC/HDL-C ratio of 4.0 or more or plasma triglycerides of 89–442 mg/dL [142]. The total sample included 2131 participants with previous cardiovascular disease and 7664 participants without cardiovascular disease. Fenofibrate treatment for five years did not significantly affect coronary heart disease-related death or nonfatal myocardial infarction rates (−11%, primary endpoint), but it significantly reduced all cardiovascular disease events (−11%, secondary endpoint). A disproportionate treatment with statins was noted during the trial in the placebo arm; thus, it was estimated that fenofibrate was associated with a “true” relative risk reduction in cardiovascular disease events by 17–20% [142]. 

The double-blind ACCORD Lipid trial [143,144] randomized 5518 patients with type 2 diabetes to fenofibrate plus simvastatin or placebo plus simvastatin for a mean follow-up of 4.7 years. Thirty-seven percent of the sample (aged 40–79 years) had established cardiovascular disease, the rest of the sample (aged 55–79 years) had evidence of subclinical cardiovascular disease or at least two additional cardiovascular risk factors and about 60% were taking a statin before enrollment. Patients were eligible if they had LDL-C levels of 60–180 mg/dL, HDL-C ≤55 mg/dL for women and blacks or ≤50 mg/dL for all other groups and triglycerides ≤750 mg/dL if they were not receiving lipid therapy or ≤400 mg/dL if they were receiving lipid therapy. Fenofibrate on top of simvastatin treatment significantly reduced total cholesterol and triglyceride levels and increased HDL-C concentration but did not significantly reduce the primary outcome (first occurrence of nonfatal myocardial infarction, nonfatal stroke or death from cardiovascular disease), secondary outcomes or death from all causes. In a prespecified analysis, fenofibrate significantly reduced the primary outcome rate in the subgroup of patients with high TG levels (≥204 mg/dL) and low HDL-C concentration (≤34 mg/dL). 

Thus, there is compelling evidence that the beneficial cardiovascular effects of fibrates (mainly fenofibrate) are mainly observed in the subgroups with diabetes and mixed dyslipidemia [143,144,145]. A meta-analysis of 18 trials based on the data from 45,058 participants reported a significant 10% relative risk reduction for major cardiovascular events and a 13% relative risk reduction for coronary events with fibrate therapy, with stronger effects being observed in groups with high triglyceride levels [146].

Worth mentioning, fenofibrate has been correlated with beneficial effects on diabetic microvascular complications. In the ACCORD Lipid study, a lower rate of incident albuminuria and a slower estimated glomerular filtration rate decline was observed with fenofibrate [147]. In the ACCORD eEye study, the rate of progression of diabetic retinopathy was reduced in the fenofibrate group by 40% [148].

More recently, a newer fibrate, pemafibrate, reached phase III clinical trials. Pemafibrate is a selective PPARα modulator (SPPARMα), with more pronounced effects on triglycerides and HDL-C levels and fewer side effects compared with other fibrates [149]. However, the development program for cardiovascular disease prevention was terminated based on the findings of the double-blind placebo-controlled PROMINENT clinical trial, where a planned interim analysis showed that it was unlikely for the primary endpoint of cardiovascular benefit to be met [150]. The PROMINENT study included 10,497 statin-treated patients with type 2 diabetes (66.9% with established cardiovascular disease), triglyceride levels of 200–499 mg/dL and HDL-C levels ≤40 mg/dL. Pemafibrate treatment for a median follow-up of 3.4 years significantly reduced triglycerides, VLDL cholesterol, remnant cholesterol (cholesterol transported in triglyceride-rich lipoproteins after lipolysis and lipoprotein remodeling) and APOC3 when given on top of statin in patients with established cardiovascular disease or patients with diabetes and cardiovascular risk factors. However, it did not reduce apolipoprotein B (APOB) concentration, which represents the number of atherosclerotic particles in the circulation, a finding that may explain the nonsignificant effects on CVD risk [150] (Table 3).

Although pemafibrate did not reduce residual cardiovascular risk in the PROMINENT study, it has some other promising effects. Pre-clinical and clinical evidence point to beneficial effects on nonalcoholic fatty liver disease (NAFLD) and nonalcoholic steatohepatitis (NASH) suggesting that pemafibrate can delay progression of fibrosis and prevent complication such as cirrhosis, hepatocellular carcinoma and liver failure [151]. In a retrospective multicenter study of 138 NAFLD patients pemafibrate improved the levels of aspartate aminotransferase (AST), alanine aminotransferase (ALT) and gamma-glutamyl transferase (GGT), fibrotic biomarkers and FAST score (FibroScan-AST), a novel index of steatohepatitis [152]. Another retrospective 24-week study reported that pemafibrate reduced liver shear wave velocity and the levels of ALT, AST, γ-GT and triglycerides, whereas HDL-C and platelet count were significantly increased [153]. A double-blind, placebo-controlled, phase 2 trial included 118 patients with liver fat content of ≥10% by magnetic resonance imaging-estimated proton density fat fraction (MRI-PDFF), liver stiffness of ≥2.5 kPa by magnetic resonance elastography (MRE) and elevated ALT levels. No significant difference was observed between pemafibrate and placebo in liver fat content (primary endpoint). However, pemafibrate reduced ALT and LDL-C levels; additionally, MRE-based liver stiffness significantly decreased compared to placebo at week 48, an effect that was maintained at week 72 [154].

Notably, NAFLD and NASH have been proposed to contribute to cardiovascular risk directly and/or as an expression of ectopic fat accumulation [155,156,157] Thus, the possible beneficial effects of pemafibrate on liver fat accumulation (and possibly to other ectopic fat compartments?) may be associated with a reduction in cardiovascular risk.

In the era of precision medicine, the use of the SPPARMα, pemafibrate, may help patients with high triglyceride levels to reduce the risk of pancreatitis, patients with metabolic syndrome to improve their metabolic profile and patients with NAFLD to reduce liver fat content [158]. A certain advantage of pemafibrate is its hepatic metabolism and excretion into the bile, in contrast with most of the fibrates which are mainly excreted from the kidney; thus, pemafibrate can be used rather safely even in patients with chronic kidney disease [159].

The FDA approved indications of fibrates are as follows [160]:

- For use as an adjunct to dietary modifications (restricted in saturated fats and cholesterol) in adults with primary hypercholesterolemia or mixed dyslipidemia (Fredrickson type IIa and IIb). Fibrates help to reduce serum LDL, total cholesterol, triglycerides, APOB and increase HDL-C.
- To be used as an adjunct to dietary modifications in adults with severe hypertriglyceridemia (Fredrickson type IV and V).

According to the ESC/EAS guidelines, fenofibrate or bezafibrate may be considered in combination with a statin in primary prevention or high-risk patients who are at LDL-C goal with triglyceride levels greater than 200 mg/dL, as well as for the prevention of recurring pancreatitis due to severe hypertriglyceridemia (i.e., plasma triglyceride levels over 500 mg/dL) [161].

Given the inverse correlation between plasma HDL-C and triglyceride levels [49], fibrates could be considered for the effective increase in HDL-C levels in men with HDL-C below 40 mg/dL or women with HDL-C below 50 mg/dL, even if their plasma triglyceride levels are normal. Properly designed clinical trials may provide conclusive answers to this question.

## 6. Conclusions

PPARα appears to be an important regulator of various apolipoproteins and lipoprotein metabolic enzymes, thus affecting the levels and functionality of plasma lipoproteins. Fibrates have a significant undisputed advantage in the treatment of severe hypertriglyceridemia, though the benefits in cardiovascular morbidity may be limited to patients with high triglyceride and low HDL-C levels. Certainly, fibrates have been very successful medicines for the prevention of pancreatitis in patients with extremely high triglyceride levels. Nevertheless, the development of new molecules has been based on outdated knowledge dating many decades back.

Even though PPARα activation influences numerous metabolic molecular pathways, the precise mechanism(s) of action remains vague. Since activated PPARα is a transcription factor affecting the expression of all genes containing PPREs (i.e., many hundreds of genes), it remains unclear which of the affected pathway(s) may be more relevant in human atherosclerosis. Moreover, the differential and often conflicting effects triggered by different agonists in clinical trials indicate that either different PPARα–fibrate complexes have different specificity for PPREs, or different fibrates have off-target effects that dictate to a great extend their clinical performance. Given that gene expression is regulated by multiple transcription factors, it is possible that the affinity of the activated PPARα agonist for PPREs in the context of all other transcription factors may dictate which genes will be activated/inhibited and to what extent. For example, a moderate activation or inhibition of a key pathway may be more therapeutic than a potent one. To address these possibilities, extensive additional research is needed to further clarify those important molecular pathways leading to atheroprotection in response to PPARα activation. In turn, this may further facilitate the creation of new and more efficient PPARα agonists which may differentially and selectively activate those pathways that matter the most. This lack of basic knowledge creates a critical gap in the understanding of PPARα physiology that cannot be overcome by the most recent pharmacological efforts to design new pan-agonists or mixed α-, γ- and/or δ- agonists.

The precise mechanisms for the multiple beneficial pharmacological effects of PPARα agonists are very complex, may involve direct and indirect events and need to be further investigated. Notably, plasma triglyceride levels show an inverse correlation with plasma HDL-C levels and additional pharmacological benefits of fibrates such as raising low HDL-C levels to physiological should be considered.

## Figures and Tables

**Table 1:** PPARα and PPARγ binding sites of various genes involved in lipid metabolism, determined by in silico analysis using Molotool software v2.0 (HOCOMOCOv11) (https://molotool.autosome.org/ accessed on 13 February 2023).

Gene	NCBI Ref. Sequence for Transcription Start	Start	End	Strand	PPAR	Sequence of the PPRE	log10 (*p*-Value)
APOA1	NM_000039.3	−517	−553	-	PPARα	AACCTGGGGAGAGGGGA	4.082
		−517	−553	-	PPARγ	AACCTGGGGAGAGGGGA	4.410
		−426	−442	+	PPARγ	GGGCGGGGGAAGGGGGA	4.065
		−186	−202	-	PPARα	CTGCAGGGCAGGGGTCA	5.041
		−186	−202	-	PPARγ	CTGCAGGGCAGGGGTCA	5.474
APOA2	NM_001643.2	−720	−736	-	PPARα	TACCAGGGTAAAGGTTG	4.073
		−397	−413	+	PPARα	AAGTGGGATAAGGTTGA	4.181
		−397	−413	+	PPARγ	AAGTGGGATAAGGTTGA	4.043
		−80	−96	-	PPARα	CAGTGGGGCAGGGATTA	4.238
		−80	−96	-	PPARγ	CAGTGGGGCAGGGATTA	4.363
APOA5	NM_001371904.1	−771	−787	+	PPARα	GGGAAGGTTAAAGGTCA	4.199
		−771	−787	+	PPARγ	GGGAAGGTTAAAGGTCA	4.640
		−478	−494	+	PPARα	AGCTGGGGCAGAGGGAT	4.078
		−478	−494	+	PPARγ	AGCTGGGGCAGAGGGAT	4.536
APOC3	NM_000040.3.	−641	−657	-	PPARα	GTGTAGGGCAGGGGTTG	4.073
		−641	−657	-	PPARγ	GTGTAGGGCAGGGGTTG	4.16
		−65	−81	-	PPARγ	GCGCTGGGCAAAGGTCA	4.069
APOE	NM_001302691.2	−125	−141	-	PPARγ	CAGCAGGGCAGAGGGAG	4.333
ME2	AC011481	70	78	+	PPARγ	CAGAGGGGA	3.681
APOM	NM_019101.3	−21	−37	+	PPARγ	GAAAGGGTCAAGGGTCG	4.086
Lpl	NM_000237.3	−169	−153	-	PPARα	AAGAGGGGGAAAGGGCA	5.901
		−169	−153	-	PPARγ	AAGAGGGGGAAAGGGCA	6.662
CETP	NM_000078.3	−675	−691	-	PPARα	ATCCGGGGGAAAGGGGC	4.259
		−675	−691	-	PPARγ	ATCCGGGGGAAAGGGGC	4.406
SRB1	NM_005505.5	−328	−344	+	PPARα	AGGTGGGGGAAGGGGTA	4.700
		−328	−344	+	PPARγ	AGGTGGGGGAAGGGGTA	4.869
		−316	−332	+	PPARα	GGGTAGGAGAAAGGGGA	4.190

APOA1: apolipoprotein A1; APOA2: apolipoprotein A2; APOA5: apolipoprotein A5; APOC3: apolipoprotein C3; APOE: apolipoprotein E; APOM: apolipoprotein M; ME2: multienhancer 2; LpL: lipoprotein lipase; CETP: cholesteryl ester transfer protein; SRB1: scavenger receptor class B type 1; PPARα: peroxisome proliferator-activated receptor α; PPARγ: peroxisome proliferator-activated receptor γ; PPRE: PPAR response element. Transcription start for various genes was considered relative to exon 1 of a variant for which the NCBI Reference Sequence was noted in the second column of the table. The statistic score that attributes a PPRE to a sequence is in the last column. High scores (above 4.5) are in blue and low scores (below 4.5) are in green. The consensus sequence used by Molotool software for the detection of the binding sites for PPARα is hWbKRGGbbARAGGKYR, and for PPARγ is vWbbRGGbSARAGGKSR.

**Table 2:** Fibrates in everyday clinical practice.

Pharmacology	Important Points
Pharmacokinetics	Well absorbed by the gastrointestinal tract. Associated with plasma proteins. Excreted through the urine, either unchanged or in the form of glucuronide metabolites. Have the potential to displace warfarin from its binding proteins and trigger an enhanced hypoprothrombinemic effect associated with prolonged bleeding. Mild to moderate inhibitors of CYP2C9.
Adverse effects	Myositis and rarely rhabdomyolysis, when administered in combination with a statin in elderly patients with many comorbidities or patients with impaired renal function (rare). Contraindicated in patients with severely impaired hepatic and renal function.
Pharmacological benefit	Reduction in plasma triglyceride levels by 20–50% *. Reduction in plasma total cholesterol by 10–15%. Increase in HDL-C by up to 20% *.

* Dependent on baseline levels. CYP2C9: cytochrome P450 family 2 subfamily C member 9; HDL-C: high-density lipoprotein cholesterol.

**Table 3:** Most important outcomes from fibrates clinical trials.

Agent	Trial	Clinical Outcomes
Bezafibrate	***BIP*** [141]	Reduced fatal myocardial infarction, nonfatal myocardial infarction and sudden death by 9%. Reduced myocardial infarction by 39% and nonfatal myocardial infarction by 33% in patients with metabolic syndrome. Reduced cardiac mortality by 66% in the subgroup of patients with 4 or 5 features of the metabolic syndrome.
Gemfibrozil	***Helsinki Heart Study*** [139]	Reduced fatal and nonfatal myocardial infarction events by 34% and total cardiovascular events by 56%, in men with hypertriglyceridemia. Reduced incidence of coronary heart disease events by 71% in the high-risk subgroup with LDL-C/HDL-C ratio >5 and triglycerides concentration >203 mg/dL
	***VA-HIT*** [140]	Reduced coronary heart disease death, nonfatal myocardial infarction or stroke by 24%. Reduced coronary heart disease events by 22%, stroke by 25% and coronary heart disease-related death by 22%.
Fenofibrate	***FIELD*** [142]	Reduced all cardiovascular disease events by 11% in patients with diabetes (estimated true reduction by 17–20% due to statin treatment).
	***ACCORD*** [143,144]	Reduced total cholesterol and triglyceride levels and increased HDL-C concentration when on top of simvastatin. Reduced first occurrence of nonfatal myocardial infarction, nonfatal stroke or death from cardiovascular disease in the subgroup of patients with high triglyceride levels (≥204 mg/dL) and low HDL-C concentration (≤34 mg/dL).
Pemafibrate	***PROMINENT*** (terminated) [150]	Reduced triglycerides, VLDL-C, remnant cholesterol and APOC3 when on top of statin in patients with established cardiovascular disease or patients with diabetes and cardiovascular risk factors. However, it did not decrease cardiovascular risk, and as a result its cardiovascular disease prevention program development was terminated.

LDL-C: low-density lipoprotein cholesterol; HDL-C: high-density lipoprotein cholesterol; VLDL-C: very low-density lipoprotein cholesterol; APOC3: apolipoprotein C3.

## Data Availability

Not applicable.
